# Morphological Ambiguity between *Chambersiella* Cobb, 1920, and *Geraldius Sanwal*, 1971: A Taxonomic Dilemma Solved through a Note from N. A. Cobb

**DOI:** 10.2478/jofnem-2025-0051

**Published:** 2025-11-05

**Authors:** Jonathan D. Eisenback, Paulo Vieira

**Affiliations:** School of Plant and Environmental Sciences, Virginia Tech, Blacksburg, VA 24061; Mycology and Nematology Genetic Diversity and Biology Laboratory, United States Department of Agriculture, Agricultural Research Service, Beltsville, MD 20705

**Keywords:** *Chambersiella galapagoensis* n. comb., *Chambersiella inserrai* n. comb., *Chambersiella jejuensis* n. comb, *Chambersiella*, Chambersiellidae, generic synonymy, *Geraldius*, monodelphic vs. didelphic, N. A. Cobb, nematode morphology, ovary number, taxonomy

## Abstract

The genera *Chambersiella* and *Geraldius* (Nematoda) are nearly morphologically identical, differing primarily in female ovary number: *Chambersiella* was described as monodelphic, while *Geraldius* was diagnosed as didelphic. This note reevaluates the validity of that distinction, incorporating original descriptions and a previously overlooked archival note from N. A. Cobb. Field observations failed to recover monodelphic specimens, even in type localities. We propose synonymizing *Geraldius* with *Chambersiella*, supported by Cobb’s archived observations and consistent morphological evidence.

Accurate delimitation of nematode genera is essential for biodiversity assessments and ecological studies. *Chambersiella* (Cobb, 1920) and *Geraldius* (Sanwal, 1971) are two morphologically similar genera within the family Chambersiellidae. Their distinction is based solely on the number of ovaries in females – a character whose diagnostic value is here reconsidered. This note integrates classical descriptions, field collections, and archival notes from Cobb to reexamine the status of these genera.

## Materials and Methods

**Figure 1:**
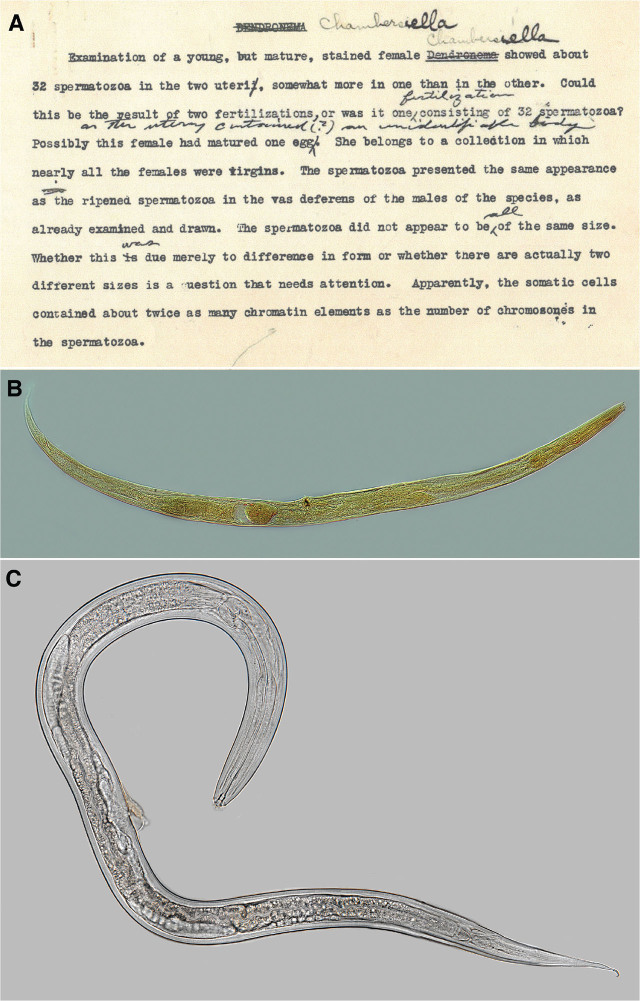
(A) Typewritten note by N. A. Cobb and filed with the type specimen of *Chambersiella rodens* Cobb, 1920 pointing out that the specimen had two uteri. (B) Photomicrograph of a female of *Chambersiella* sp. from entry number 11034, slide G-1671, from the USDANC, collected by J. Saunders via Gerald Thorne or Mae Noffsinger from the beetle gallery of *Scolytus mutistriatus* Marsham, 1802 showing a didephic ovary. (C) Photomicrograph of a female of *Chambersiella rodens* Cobb, 1920 collected from a seed cone of Japanese cedar, *Cryptomeria japonica* (L. f.) D. Don.

We reviewed the original descriptions of *Chambersiella rodens* Cobb, 1920 (Cobb, 1920) and *Geraldius bakeri* Sanwal, 1971 (Sanwal, 1971), and a typewritten note by N. A. Cobb preserved in the USDA Nematode Collection, Beltsville, MD (Fig. 1). Unfortunately, the type specimen could not be found. Comparisons focused on ovary number, the sole feature used to differentiate the genera. Morphological data were compiled from published illustrations, morphometrics, and direct field observations.

Additionally, specimens were collected from the tree bark of pine (*Pinus* spp.) and oak (*Quercus* spp.), and Spanish moss, *Tillandsia usneoides* (L.) L. in Alabama, Florida, Georgia, Maine, New Hampshire, North Carolina, South Carolina, Tennessee, Vermont, and Virginia.

## Results and Discussion

Cobb’s original description includes the phrase, “The gently tapering ovary contains 50–100 ova; reflexed to near anus,” suggesting a posteriorly reflexed second ovary. This interpretation is confirmed by a note from Cobb stating, “Examination of a young, but mature, stained female *Chambersiella* showed about 32 spermatozoa in the two uteri, somewhat more in one than the other” (Fig. 1A). If Cobb observed “spermatozoa in two uteri,” this suggests that either the female was didelphic (most likely), or the second structure was mistakenly called a uterus, but could have been a seminal receptacle, uterine diverticulum, or an abnormality.

Only two slides of *Chambersiella* sp. were found in the USDANC from entry number 11034, slides G-1671 and G-1672, collected by J. Saunders via Gerald Thorne or Mae Noffsinger from the beetle gallery of *Scolytus mutistriatus* Marsham, 1802, which shows a didelphic ovary (Fig. 1B). Unfortunately, the type specimen was not found.

All specimens that were collected along the eastern coast of the United States from both oak and pine trees were all didelphic. No monodelphic specimens were recovered, even in areas where *C. rodens* was originally reported. Measurements (Table 1) and photomicrographs (Fig. 1C) of specimens identified by the authors as *C. rodens* are presented here as further support that *Chambersiella* is didelphic.

**Table 1:** Morphometric of females of *Chambersiella rodens* Cobb, 1920, *C. inserrai* Cid del Prado et al., 2021 *C. bakeri* Sanwal, 1957, *C. galapagoensis* Cid del Prado, 2012 and *C. jejuensis* Mwamula et al., 2025 from the original descriptions, additional measurements of *C. bakeri* by Holovachov, and original measurements of *C. rodens* by Eisenback and Vieira.

**Character**	***C. rodens* (Cobb, 1920)**	***C. rodens* (this study)**	***C. inserrai* (Cid del Prado et al., 2021)**	***C. bakeri* (Sanwal, 1957)**	***C. bakeri* (Holovachov et al., 2003)**	***C. galapagoensis* (Cid del Prado et al., 2021)**	***C. jejuensis* (Mwamula et al., 2025)**
*n*	1	5	5	–	10	19	16
L	(670)	905–1,354 (1,077)	1,000–1,200 (1,100)	1,100–1,300 (1,200)	1,001–1,200 (1,100)	800–1,200 (1,000)	1,040–1,306 (1,163)
a	(27.0)	19.6–24.0 (21.4)	24.6–28.8 (26.9)	–	17.6–23.5 (20.7)	19.3–35 (25.0)	23.6–30.3 (26.9)
b	(5.0)	4.1–5.8 (4.6)	4.5–5.1 (4.9)	4.4–5.3 (4.9)	4.4–5.3 (4.9)	4.9–6.8 (5.7)	4.9–5.9 (5.4)
c	(15.6)	7.2–10.7 (9.0)	8.9–14.5 (10.3)	8.0–9.5 (8.8	8.0–9.5 (8.9)	8.1–10.8 (9.6)	7.0–7.9 (7.4)
c′	(2.1)	3.7–5.4 (4.6)	3.3–5.8 (5.0)	4.1–6.3 (5.2)	4.1–6.3 (5.1)	3.7–6.3 (4.7)	5.7–7.2 (6.3)
V%	(58)	42.0–53.3 (51.1)	47.3–53.9 (49.9)	48.8–52.3 (50.0)	48.8–52.3 (50.2)	39–50 (47)	46.8–49.3 (48.0)
Esophagus l.	(135.3)	218.5––262.0 (235.9)	215–247 (228.0)	224–248 (236)	224–248 (238.0)	141–207 (179)	207.0–227.0 (217.2)
Corpus	–	130.9––136.3 (135)	134–143 (139.4)	120–138 (129)	120–138 (131)	112–145 (125)	123.5–140.0 (131.4)
Excretory pore	–	163.2––202.0 (178.7)	155–185 (16(6.5))	171–190 (180)	171–190 (178)	73–161 (133)	148.0–183.0 (164.9
Tail length	(42.9)	102.0––142.0 (120.4)	116–127 (121.3)	121–144 (132)	121–144 (131)	81–121 (105)	146.0–177.0 (156.4)
BDA	(20.8)	25.0––28.8 (27.0)	21–27 (22.6)	23–31 (27)	23–31 (26)	18–28 (24.4)	22.5–27.0 (24.8)
Ant. to NR	(85.1)	140.1––151.0 (146.1)	145–166 (154.5)	–	144–165 (157)	110–145 (130.5)	131.0–155.5 (145.0)
Phasmid to anus	–	27.1––43.0 (37)	40–47 (43.2)	33–46 (39)	33–46 (39.8)	32–51 (40.5)	55.0–78.5 (64.8)
Ant. to deirid	–	–	154–166 (159.0)	166–190 (178)	166–190 (178)	129–152 (138)	149.0–183.0 (165.4)
BD at vulva	(24.8)	43.6––58.9 (48.2)	42–50 (45.6)	–	–	35–64 (49)	33.5–45.5 (40.4)
BD maximum	(24.8)	45.9––60.8 (50.1)	38–48 (41.4)	46–67 (57)	46–67 (55.8)	30–55 (41)	38.0–48.0 (43.3)

BD, maximum body diameter; BDA, body diameter at anus; NR = nerve ring.

After more than 100 years, *Chambersiella* remains monotypic, whereas *Geraldius* contains at least five species (Cobb, 1920; Sanwal, 1957; Cid del Prado, 2012; Cid del Prado et al., 2021; Mwamula et al., 2025). These findings cast doubt on the use of ovary number as a valid generic character and suggest that the genera are not distinct. The cardial morphology, cephalic cirri, and esophageal anatomy are otherwise indistinguishable between the two groups.

Preliminary molecular studies on *Geraldius inserrai* Cid del PradoVera et al., 2021 and *G. jejuensis* Mwamula et al., 2025 (Cid del Prado et al., 2021; Mwamula et al., 2023) suggest close phylogenetic affinity with other *Geraldius* species, lending support to their inclusion within the same genus. If *Chambersiella* does indeed have two ovaries, it has not been reported since its description; therefore, molecular studies cannot be completed on nematode that cannot be found. Moreover, integrative taxonomic frameworks employing multiple loci and species delimitation methods (e.g., General Mixed Yule-Coalescent [GMYC] (Zhang et al., 2013), Automatic Barcode Gap Discovery [ABGD] Puillandre et al., 2012), or Bayesian Poisson Tree Processes [bPTP]) can further substantiate the collapse of these two genera into a unified *Chambersiella* sensu lato (Derycke et al., 2010).

Therefore, the integration of classical morphology, historical taxonomic intent (Cobb, 1920), and modern molecular diagnostics represents a comprehensive path forward for resolving this generic controversy in Chambersiellidae.

## Conclusion

The historical separation of *Chambersiella* and *Geraldius* based solely on ovary number is undermined by Cobb’s archived notes and extensive field observations. We propose that *Geraldius* is a junior synonym of *Chambersiella*, and we formally transfer the following species: *Geraldius bakeri*; *G. galapagoensis* Cid del Prado, 2012; *G. inserrai*; and *G. jejuensis* to *Chambersiella*.

## Proposed Taxonomic Changes

*Chambersiella* Cobb, 1920 syn. *Geraldius* Sanwal, 1971
Type species: *Chambersiella rodens*
*Chambersiella bakeri* Sanwal, 1957
*Chambersiella galapagoensis.* (Cid del Prado, 2012) n. comb.
*Chambersiella inserrai* (Cid del Prado et al., 2021) n. comb.
*Chambersiella jejuensis* (Mwamula et al., 2025) n. comb.

## References

[j_jofnem-2025-0051_ref_001] Cid del Prado V. I. (2012). Two new species of nematodes (Cephalobida: Chambersiellidae) from moss from North and South America. Nematropica.

[j_jofnem-2025-0051_ref_002] Cid del Prado V. I., Ferris H., Subbotin S. A. (2021). A new species of *Geraldius inserrai* sp. n (Rhabditida: Chambersiellidae) from Mexico. Nematropica.

[j_jofnem-2025-0051_ref_003] Cobb N. A. (1920). One hundred new NEMAS. Contributions to a Science of Nematology.

[j_jofnem-2025-0051_ref_004] Derycke S., De Ley P., Tandingan De Ley I., Holovachov O., Rigaux A., Moens T. (2010). Linking DNA sequences to morphology: Cryptic diversity and population genetic structure in marine nematodes. Molecular Phylogenetics and Evolution.

[j_jofnem-2025-0051_ref_005] Holovachov O., Esquivel A., Bongers T. (2003). Free-living nematodes from nature reserves in Costa Rica. 4. Cephalobina. Nematology.

[j_jofnem-2025-0051_ref_006] Mwamula A. O., Bae C. H., Lee D. G., Kim Y. S., Lee Y. D., Lee D. (2025). Description and molecular characterization of *Geraldius jejuensis* n. sp. (Nematoda: Chambersiellidae) from Korea. Journal of Nematology.

[j_jofnem-2025-0051_ref_007] Puillandre N., Lambert A., Brouillet S., Achaz G. (2012). ABGD, automatic barcode gap discovery for primary species delimitation. Molecular Ecology.

[j_jofnem-2025-0051_ref_008] Sanwal K. C. (1957). Chambersiellidae n. fam. (Nematoda) with emended diagnosis of the genus *Chambersiella* Cobb, 1920, description of *C. bakeri* n. sp., and discussion of taxonomic position. Canadian Journal of Zoology.

[j_jofnem-2025-0051_ref_009] Sanwal K. C. (1971). *Geraldius* n. gen., Macrolaiminae n. subfam., with a revision of the subfamilies and genera of Chambersiellidae (Nematoda). Canadian Journal of Zoology.

[j_jofnem-2025-0051_ref_010] Zhang J., Kapli P., Pavlidis P., Stamatakis A. (2013). A general species delimitation method with applications to phylogenetic placements. Bioinformatics (Oxford, England).

